# Diagnosing Sepsis in Primary Care: Navigating Clinical Uncertainty Across Vulnerable Patient Groups

**DOI:** 10.7759/cureus.109461

**Published:** 2026-05-22

**Authors:** Omar Elbaroumi

**Affiliations:** 1 General Practice, NHS Independent Practice, Colchester, GBR

**Keywords:** atypical presentation, clinical recognition, diagnosis, frailty, general practice, patient safety, primary care, qsofa, sepsis, sepsis recognition

## Abstract

Sepsis is a medical emergency characterised by life-threatening organ dysfunction arising from a dysregulated host response to infection. It represents one of the most significant patient safety challenges in the United Kingdom, responsible for tens of thousands of preventable deaths each year, with a growing burden of hospital admissions that has risen substantially over the past two decades. The majority of cases originate in the community, placing general practitioners (GPs) at the critical frontline of early recognition. Despite this, the evidence base for sepsis diagnosis in primary care is sparse, and widely used clinical tools, including the quick Sequential Organ Failure Assessment (qSOFA) score and the National Early Warning Score 2 (NEWS2), were developed and validated predominantly in hospital settings, limiting their direct applicability in general practice. This narrative review examines the diagnostic challenges faced by UK GPs in recognising sepsis, with a particular focus on vulnerable patient groups, including elderly and frail patients, children, and individuals with multimorbidity, who frequently present with atypical features that do not satisfy conventional diagnostic criteria. Published literature from 2015 to 2025 was reviewed alongside current UK guidelines, including the updated National Institute for Health and Care Excellence (NICE) guidelines on suspected sepsis (NG253, NG254, NG255) and UK Sepsis Trust resources. Evidence suggests that the majority of GPs rely primarily on clinical intuition rather than validated screening tools, with gut feeling cited as the principal diagnostic method by up to 98% of practitioners, while formal tools such as qSOFA are used by fewer than 10%. Awareness of the UK Sepsis Trust criteria remains particularly low. Barriers to timely diagnosis include short consultation times, the low baseline prevalence of sepsis within the undifferentiated GP caseload, the increasing use of remote and telephone consultations, and limited access to point-of-care testing. The updated NICE guidance reasserts the primacy of clinical judgement alongside structured risk stratification, yet implementation in primary care settings remains inconsistent. This review concludes that sepsis recognition in UK general practice is a high-stakes diagnostic challenge compounded by atypical presentations in vulnerable patients and systemic barriers to timely assessment. Greater investment in GP-specific training, improved awareness of current guidelines, and the development of primary care-validated diagnostic frameworks are essential to reducing preventable sepsis-related mortality originating in the community.

## Introduction and background

Introduction

Sepsis is defined as a life-threatening organ dysfunction caused by a dysregulated host response to infection [1]. It remains one of the most pressing patient safety challenges in modern medicine, claiming an estimated 48,000 lives annually in the United Kingdom and affecting approximately 245,000 people each year [2]. Despite decades of awareness campaigns, updated clinical guidelines, and significant investment in hospital-based detection systems, sepsis continues to be missed, misdiagnosed, or recognised too late, with delays in or failure to diagnose sepsis featuring in 81% of clinical negligence claims related to the condition in general practice [3].

In practical terms, sepsis occurs when the body's response to an infection begins to damage its own tissues and organs, a process that can progress rapidly from an apparently minor illness to life-threatening organ failure within hours. Unlike hospital settings, where patients are monitored continuously, investigations are available within minutes, and escalation pathways are immediately accessible, general practice operates under fundamentally different constraints: consultations typically last 10 minutes, point-of-care testing is limited, and the general practitioner (GP) must identify the rare seriously ill patient among a caseload dominated by self-limiting illness. Critically, sepsis is not primarily a hospital problem. Between 70% and 80% of sepsis cases are estimated to originate in the community, making the GP a pivotal, and often underappreciated, first point of contact in its recognition and early management [4]. An acutely unwell patient presenting to a GP surgery may be in the early stages of sepsis, yet the clinical environment of primary care differs fundamentally from the emergency department or acute medical unit where most diagnostic tools and care pathways have been developed and validated. GPs operate within time-constrained consultations, with limited access to point-of-care investigations, and face the daily challenge of identifying the rare, genuinely dangerous patient among a caseload dominated by self-limiting illness. As a recent Lancet Primary Care viewpoint noted, sepsis is generally considered a hospital-based issue, yet it usually begins in the community, where knowledge is scarce, diagnosis is difficult, and resources vary [5].

The epidemiological burden is worsening. Sepsis-coded hospital admissions in England increased more than sevenfold between 1998 and 2023, rising from 27.9 to 210.4 admissions per 100,000 population, with the proportion of patients aged 75 years and over now accounting for more than half of all admissions [6]. This demographic shift has direct implications for general practice: the patients most at risk of sepsis, the elderly, the frail, and those with multiple long-term conditions, are the very patients most likely to present with atypical, non-specific features that do not satisfy conventional diagnostic thresholds. Severe frailty, for example, has been shown to carry a crude odds ratio of nearly 15 for developing community-acquired sepsis [7].

The clinical tools currently recommended for sepsis recognition have limited applicability in primary care. The quick Sequential Organ Failure Assessment (qSOFA) score and the National Early Warning Score 2 (NEWS2) [8] were developed and validated predominantly in hospital and pre-hospital emergency settings. Survey evidence consistently demonstrates that the majority of GPs rely on clinical intuition rather than formal screening tools, with gut feeling cited as the principal diagnostic method by up to 98% of practitioners, while qSOFA is used by fewer than 10% and the UK Sepsis Trust criteria by fewer than 2% [9]. This is not simply a training failure: it reflects a genuine mismatch between tools designed for high-acuity environments and the undifferentiated, low-prevalence diagnostic context of general practice.

The regulatory and guideline landscape has evolved. The National Institute for Health and Care Excellence (NICE) guideline on suspected sepsis (NG51), first published in 2016 and substantially updated in January 2024, has now been replaced by three dedicated guidelines covering adults aged 16 and over (NG253), under-16s (NG254), and pregnant or recently pregnant people (NG255) [10]. The UK Sepsis Trust has updated its GP-specific screening tools accordingly. These developments are welcome, yet the fundamental diagnostic challenge for GPs, how to identify sepsis early, reliably, and safely within the constraints of routine primary care, remains incompletely addressed.

This narrative review aims to synthesise current evidence on the diagnostic challenges faced by UK GPs in recognising sepsis, with particular attention to vulnerable patient groups including elderly and frail patients, children, and individuals with multimorbidity. It critically evaluates the available screening tools and their limitations in primary care, examines the systemic and individual barriers to timely recognition, and proposes practical approaches to improving sepsis detection at the community level. In doing so, it argues for a shift in how sepsis diagnostic frameworks are developed and implemented, away from adapted hospital tools and towards frameworks designed with, and for, general practice.

Methods

This article is a narrative review and does not follow systematic review methodology or Preferred Reporting Items for Systematic Reviews and Meta-Analyses (PRISMA) reporting guidelines. A narrative approach was selected as appropriate given the heterogeneity of study designs, settings, and outcomes in the available literature on sepsis recognition in primary care, which precludes meaningful pooled synthesis. No formal meta-analysis was conducted.

A structured literature search was conducted using PubMed and Google Scholar. Search terms included "sepsis primary care", "sepsis general practice", "sepsis recognition community", "sepsis diagnosis elderly", and "sepsis children primary care". Searches were limited to English-language publications from 2015 to 2025, with exceptions made for seminal works predating this period, including the original systemic inflammatory response syndrome (SIRS) consensus definition [11] and the Sepsis-3 task force publications [1]. Relevant UK clinical guidelines, National Health Service (NHS) policy documents, and resources from the UK Sepsis Trust and the Royal College of General Practitioners were reviewed alongside the peer-reviewed literature. Studies and guidance pertaining to the diagnosis, recognition, and management of sepsis in pre-hospital or community settings were prioritised, with a focus on the UK healthcare context. Reference lists of the included articles were reviewed for additional relevant sources. Given the narrative design, no formal risk-of-bias assessment or study quality appraisal was conducted; the weight given to individual studies reflects their relevance to the primary care context and the directness of their applicability to the review questions.

## Review

Defining sepsis: from SIRS to Sepsis-3 and what it means for GPs

A Brief History of Sepsis Definitions

The definition of sepsis has undergone three major revisions over the past three decades, each reflecting advances in the understanding of its pathophysiology and each carrying distinct implications for clinical practice, including in primary care.

The first formal consensus definition, published in 1991, introduced the concept of the SIRS [11]. Sepsis was defined as SIRS in the presence of a known or suspected infection. SIRS was operationalised using four readily measurable clinical parameters: temperature above 38°C or below 36°C, heart rate greater than 90 beats per minute, respiratory rate greater than 20 breaths per minute or a PaCO₂ of less than 4.3 kPa, and a white blood cell count greater than 12,000 or less than 4,000 cells per mm³ or more than 10% immature band forms [11]. Two or more of these criteria, in the context of infection, were sufficient to meet the definition. The appeal of SIRS lay in its simplicity: these parameters required no laboratory values beyond a full blood count and were measurable at the bedside. For a brief period, this appeared to offer a workable framework for primary care.

However, SIRS criteria proved to be poorly discriminating in clinical practice. The criteria were found to be excessively sensitive but insufficiently specific: the majority of acutely ill patients, including those with non-infectious conditions such as pancreatitis, trauma, or burns, could meet SIRS criteria without having sepsis [12]. Conversely, a significant proportion of patients with confirmed infection and organ failure did not fulfil the minimum of two SIRS criteria, yet went on to have severe and fatal outcomes [13]. The criteria were also particularly unreliable in elderly and immunocompromised patients, who may fail to mount a classical inflammatory response [12,13]. A normal temperature, heart rate, or white cell count does not exclude sepsis, a fact of direct and daily relevance in general practice.

Sepsis-3: The Current Definition

In 2016, a task force convened by the Society of Critical Care Medicine (SCCM) and the European Society of Intensive Care Medicine (ESICM) published the Third International Consensus Definitions for Sepsis and Septic Shock, universally known as Sepsis-3 [1]. This represented a fundamental reconceptualisation. Sepsis was redefined as life-threatening organ dysfunction caused by a dysregulated host response to infection, with the term "severe sepsis" eliminated entirely as redundant. Organ dysfunction became the central diagnostic criterion, operationalised using an acute increase of two or more points in the Sequential Organ Failure Assessment (SOFA) score [1]. Septic shock was defined as a subset of sepsis in which circulatory and cellular metabolic abnormalities are sufficiently profound to substantially increase mortality, identifiable clinically by the need for vasopressor therapy to maintain a mean arterial pressure of 65 mmHg or greater and a serum lactate greater than 2 mmol/L despite adequate fluid resuscitation [1].

The conceptual shift in Sepsis-3 was important: sepsis is no longer framed as an excessive inflammatory response, but rather as a pathological, dysregulated host response that causes self-injury [1]. This has implications for how clinicians think about atypical presentations, particularly in patients whose immune systems may not respond conventionally, including the elderly, those on immunosuppressive therapy, and patients with significant frailty or multimorbidity.

The Problem of Sepsis-3 for General Practice

While Sepsis-3 represents a scientifically robust definition, its primary diagnostic tool, the SOFA score, requires laboratory values including serum creatinine, bilirubin, platelet count, and PaO₂/FiO₂ ratio [1]. These are simply not available in real-time within a standard GP consultation. The SOFA score was designed and validated in intensive care settings and cannot be meaningfully calculated by a GP seeing an acutely unwell patient in a 10-minute appointment.

To address this, the Sepsis-3 task force introduced the qSOFA score as a simpler bedside tool for use outside critical care settings [1]. qSOFA comprises three criteria: a respiratory rate of 22 breaths per minute or more, an altered mental status (any Glasgow Coma Scale score below 15), and a systolic blood pressure of 100 mmHg or less. A score of 2 or more is considered positive and should prompt concern for sepsis. The advantage of qSOFA is that it requires no laboratory testing and can, in principle, be calculated during any clinical assessment. This makes it the most transferable Sepsis-3 tool to the general practice environment.

However, qSOFA has significant limitations as a screening tool. Multiple studies and meta-analyses have demonstrated that while qSOFA has high specificity, correctly identifying patients who are severely unwell, its sensitivity is low, estimated at approximately 46%, meaning that more than half of patients with sepsis may have a negative qSOFA at initial assessment [14,15]. In a primary care setting, where the clinical imperative is not to miss a diagnosis rather than to confirm one with certainty, a tool that misses over half of cases is insufficient as a standalone screen. The Surviving Sepsis Campaign itself recommends against using qSOFA as a sole screening tool for this reason, a position reaffirmed in the most recent 2026 guidelines [16]. Given its low sensitivity, qSOFA should not be used as a standalone exclusion tool in primary care, and a negative qSOFA score does not rule out sepsis and should never be used to provide false reassurance in the community setting.

By contrast, the original SIRS criteria, while non-specific, had a sensitivity of approximately 85%, making them more useful for ruling out sepsis in low-prevalence environments such as general practice, even if their lack of specificity generates a high rate of false positives [14]. Some authors have argued that neither SIRS nor qSOFA alone is adequate for primary care and that what is needed is a composite approach incorporating clinical context, vital signs, the GP's assessment of the patient's trajectory, and structured safety netting [9].

What This Means in Practice

For the UK GP, the operational reality is this: neither the full SOFA score nor qSOFA was designed for, or adequately validated in, the primary care setting. SIRS criteria are more sensitive but generate excessive false positives. Clinical intuition, informed by the patient's overall appearance, trajectory, and the GP's prior knowledge of that individual, remains the predominant diagnostic approach, as survey evidence consistently demonstrates [9]. The challenge is not simply that GPs lack awareness of formal tools but that the tools themselves were not built with general practice in mind. This mismatch is a primary driver of missed diagnoses at the community level. A further practical tension deserves acknowledgement: the trade-off between sensitivity and over-referral burden. In a low-prevalence environment such as general practice, any highly sensitive screening approach will inevitably generate a substantial number of false positives, patients who are referred or escalated on the basis of possible sepsis but who do not have the condition. For individual GPs, this creates a genuine dilemma: the imperative not to miss a life-threatening diagnosis must be balanced against the real consequences of unnecessary emergency referral, including patient anxiety, ambulance resource utilisation, and pressure on already-stretched emergency departments. There is currently no validated primary care tool that adequately resolves this tension, and its acknowledgement is essential to understanding why GPs may be reluctant to apply low-threshold screening approaches in routine practice, even when they are aware of the relevant guidance.

Diagnostic tools available to GPs: evidence and limitations

The UK Sepsis Trust Primary Care Screening Tool

In recognition of the limitations of hospital-derived tools, the UK Sepsis Trust has developed a suite of clinical screening tools specifically adapted for different healthcare settings, including general practice and out-of-hours services [17]. Updated in 2024 in response to the revised NICE guidance, the UK Sepsis Trust primary care tool guides clinicians through a structured risk stratification process using red flag and amber flag criteria. Red flags, including a non-blanching rash, mottled or ashen appearance, cyanosis, seizure activity in the context of infection, or a systolic blood pressure below 90 mmHg, should prompt immediate emergency referral. Amber flags prompt urgent same-day review and escalation if clinical concern persists.

The UK Sepsis Trust tool is notable in that it incorporates clinical context and physiological parameters accessible in a GP surgery, without requiring laboratory values. It also explicitly acknowledges the role of clinical judgement, stating that clinicians should refer any patient in whom they have a clinical concern about sepsis, even in the absence of formal red flags. Despite this, awareness of the UK Sepsis Trust tool among GPs remains low: survey evidence indicates that fewer than 12% of GPs have heard of it and fewer than 2% report using it in practice [9]. This represents a significant implementation gap, particularly given that the tool is freely available and requires no additional equipment.

NEWS2 in the Primary Care Context

The NEWS2 is the standardised physiological scoring system endorsed by NHS England and mandated across acute hospital settings, where it has been validated for deterioration detection and demonstrates strong predictive performance for sepsis-related mortality [8]. However, it is important to distinguish between three separate dimensions of its utility: diagnostic accuracy, risk stratification, and standardisation of documentation. In terms of diagnostic accuracy, NEWS2 was validated in acute inpatient populations, and its predictive performance changes substantially in community settings where baseline physiological abnormalities are common, patient acuity is lower, and the prevalence of true sepsis is far lower than in hospital cohorts [8,9]. In terms of risk stratification utility, while NEWS2 cannot be recommended as a standalone sepsis screening tool in primary care, structured measurement of its six component parameters, respiratory rate, oxygen saturation, systolic blood pressure, pulse rate, level of consciousness, and temperature, may still provide clinical benefit by prompting systematic physiological assessment and facilitating comparison with a patient's known baseline [8]. In terms of documentation, NEWS2 offers a standardised framework for recording observations that supports continuity of care and is recognised across care settings, which is of particular value when a GP is escalating a patient to secondary care. The updated NICE guidance recommends NEWS2 for use in ambulances and acute hospital settings on taking over care from the community, rather than as a primary care screening tool per se [10].

The role of clinical intuition: evidence for and against

The predominance of gut feeling in GP sepsis recognition is well documented. Survey evidence consistently shows that up to 98% of GPs cite clinical intuition as their primary diagnostic method when assessing patients with possible sepsis, with formal scoring tools used by a small minority [9]. A separate Dutch GP survey found that general appearance, patient history, and gut feeling were ranked above vital sign measurement as drivers of the decision to refer [18]. This is not merely an anecdote of undertrained practitioners: it reflects the complex, holistic, and contextual nature of diagnostic reasoning in primary care, where the GP's knowledge of the individual patient, their baseline, and their trajectory over time provides diagnostic information unavailable to any scoring system. Continuity of care, the longitudinal relationship between a GP and their registered patient population, is itself a diagnostic asset that deserves explicit recognition. A GP who has known a patient for years possesses contextual knowledge that no acute assessment tool can replicate: awareness of that patient's usual affect, functional capacity, baseline observations, and pattern of illness behaviour. This knowledge enables the detection of subtle deviation from baseline that may represent the earliest manifestation of sepsis, often before formal diagnostic criteria are met. In an era of increasing GP workload, fragmented care, and remote consultation, the erosion of continuity of care represents not only a relational loss but a genuine patient safety concern, one that is particularly acute in the context of time-sensitive conditions such as sepsis.

Importantly, research suggests that while GPs rarely use formal criteria consciously, their clinical reasoning largely mirrors qSOFA parameters. A study of GP diagnostic behaviour found that altered mental status, hypotension, and increased respiratory rate, the three qSOFA components, were the physiological signs most strongly associated with GPs' decision to refer [9]. This suggests that the intuitive process is not arbitrary, but rather an implicit application of the same physiological reasoning that underpins formal tools, without the structure or consistency that formalisation would provide. The implication is not that gut feeling should be discouraged, but that it should be supplemented by structured approaches that make implicit reasoning explicit and auditable.

Emerging primary care-specific prediction models

Recognising the absence of validated sepsis diagnostic tools for primary care, a small number of researchers have begun to develop and test primary care-specific prediction models. Loots and colleagues conducted a prospective cohort study across four out-of-hours primary care services in the Netherlands, enrolling acutely ill adults receiving home visits between 2018 and 2020 [19]. They developed a clinical prediction model incorporating nine bedside variables, including altered consciousness, tachypnoea, tachycardia, hypotension, and capillary refill time, alongside point-of-care biomarkers including C-reactive protein (CRP), procalcitonin, and lactate. The model demonstrated reasonable discriminatory performance, with an area under the receiver operating characteristic curve (AUROC) of 0.85 in the development cohort. Notably, qSOFA performed poorly in the same population, with an AUROC of only 0.65, reinforcing the argument that hospital-derived tools are poorly calibrated for primary care populations.

This work represents an important step towards a primary care-validated framework, but significant gaps remain. The study was conducted in a Dutch out-of-hours setting with home visiting capability, which differs substantially from the UK GP surgery context, where home visits are less frequent and point-of-care biomarker access is inconsistent. Generalisation to UK daytime general practice requires further prospective validation.

Point-of-care testing: CRP and lactate

Point-of-care testing offers a potential adjunct to clinical assessment in primary care, providing near-immediate biomarker results without the delay of laboratory processing. Two biomarkers are of particular relevance to sepsis recognition in general practice: CRP and lactate.

CRP is a non-specific marker of inflammation that is already widely used in UK general practice to guide antibiotic prescribing decisions in respiratory tract infections. Its role in sepsis recognition is more limited: while elevated CRP supports the presence of a significant inflammatory response, it lacks the specificity to distinguish sepsis from other causes of inflammation, and a normal CRP does not exclude early sepsis. A 2025 commentary in the British Journal of General Practice noted that while CRP point-of-care testing is recommended in national guidelines for respiratory infections and is part of NHS England commissioning specifications for urgent care, its use in UK GP out-of-hours services remains rare, and its effectiveness in this setting has not been established [20].

Lactate is a more specific marker of tissue hypoperfusion and is central to the Sepsis-3 definition of septic shock. A systematic review published in the British Journal of General Practice identified no high-quality evidence to support the use of point-of-care lactate in community settings, and no studies had been conducted in a GP out-of-hours or general practice context [21]. While trends towards reduced mortality with point-of-care lactate were observed in emergency department studies, the evidence base remains insufficient to recommend its routine use in primary care, and practical barriers, including equipment cost, training requirements, and the time needed to obtain results within a short consultation, further limit its current applicability.

Summary

The diagnostic toolkit available to UK GPs for sepsis recognition is limited and imperfectly suited to the primary care environment. Formal tools such as qSOFA and NEWS2 were designed and validated in hospital settings and carry significant sensitivity limitations when applied in primary care. The UK Sepsis Trust primary care tool offers the most contextually appropriate structured approach, but awareness and uptake remain low. Clinical intuition, while dominant and partially evidence-based, is variable between practitioners and cannot be systematically audited or improved without a structured framework. Point-of-care biomarkers show promise but lack a robust evidence base in the UK GP context. Taken together, these limitations underscore the need for a primary care-specific, prospectively validated diagnostic approach to sepsis recognition, one that integrates physiological assessment, biomarker support where available, and the contextual clinical knowledge that is the GP's unique asset. Table 1 summarises the key features, limitations, and primary care applicability of the diagnostic tools discussed in this section.

**Table 1:** Comparison of available sepsis diagnostic tools in the primary care setting GPs: general practitioners; qSOFA: quick Sequential Organ Failure Assessment; RR: respiratory rate; SBP: systolic blood pressure; HR: heart rate; SpO₂: oxygen saturation; WBC: white blood cell count; temp: temperature; NEWS2: National Early Warning Score 2; SIRS: systemic inflammatory response syndrome; UKST: UK Sepsis Trust; CRP: C-reactive protein; POCT: point-of-care testing Sensitivity and specificity data for qSOFA are derived from Serafim et al. [14], a systematic review and meta-analysis of predominantly emergency department and hospital cohorts, and Freund et al. [15], a multicentre emergency department study. These figures reflect performance in acute care settings and should be interpreted with caution when applied to primary care populations, where disease prevalence and patient acuity differ substantially. No validated sensitivity or specificity data exist for the UKST primary care tool in prospective community cohorts. The UKST tool awareness data are derived from Mulders et al. [9], a survey of Dutch GPs. The UKST tool is available at sepsistrust.org [17].

Tool	Parameters required	Lab values needed?	Sensitivity	Specificity	Validated in primary care?	Notes for GPs
qSOFA	RR ≥22, altered mentation, SBP ≤100 mmHg	No	~46%	High	No	Misses >50% of cases; not recommended as a sole screening tool
NEWS2	RR, SpO₂, SBP, HR, consciousness, temp	No	Moderate	Moderate	No	Unreliable in multimorbid patients with abnormal baseline observations
SIRS criteria	Temp, HR, RR, WBC	WBC only	~85%	Low	No	High false-positive rate; superseded by Sepsis-3 but more sensitive
UKST primary care tool	Red/amber flag criteria; clinical judgement	No	Not formally validated	Not formally validated	Yes (designed for GP)	Best available GP-specific tool; used by <2% of GPs despite being free
CRP (point-of-care)	CRP level	Yes (POCT)	Low (non-specific)	Low (non-specific)	No	Cannot distinguish sepsis from other causes of inflammation
Lactate (point-of-care)	Serum lactate	Yes (POCT)	Moderate	High (for septic shock)	No	No high-quality evidence for community use; equipment rarely available in UK GP
Clinical intuition	Clinical gestalt, patient trajectory, baseline knowledge	No	Variable	Variable	Predominant method	Used by 98% of GPs; implicit qSOFA reasoning; valuable but not auditable

Atypical presentations: the diagnostic trap in vulnerable patient groups

Perhaps the most significant clinical challenge in sepsis recognition for the UK GP is the frequency with which the condition presents atypically in the patients most likely to develop it. The classic features of sepsis, fever, tachycardia, rigors, and an obvious infective source, are a pattern that belongs more to the textbook than to the consulting room. In general practice, where the highest-risk patients are the elderly, the frail, and those with multiple comorbidities, sepsis routinely presents without these hallmarks. Understanding the specific diagnostic pitfalls in each vulnerable group is essential to reducing missed diagnoses at the community level.

The Elderly and Frail Patient

Older patients represent both the fastest-growing demographic in UK general practice and the group at greatest risk of sepsis and its complications. Data from linked primary care and hospital records in England demonstrate that severe frailty carries a crude odds ratio of nearly 15 for developing community-acquired sepsis and that severely frail patients have a case fatality rate of 42% compared to 24% in non-frail patients [7]. The incidence of sepsis increases sharply with age, and patients aged 75 and over now account for more than half of all sepsis-coded hospital admissions in England [6].

The physiological changes of ageing fundamentally alter the presentation of sepsis in this group. Immunosenescence, the progressive decline in immune function associated with ageing, may blunt or attenuate the inflammatory response that generates the classical signs of infection, meaning that fever, tachycardia, and leukocytosis, while not uniformly absent, are variable and frequently less pronounced than would be expected in younger patients [22]. Fever, the most recognisable feature of systemic infection, may be absent or present at a lower threshold. Tachycardia may be masked by beta-blocker therapy, which is widely prescribed for cardiovascular conditions in this age group and which prevents the compensatory heart rate rise that would otherwise serve as an early warning sign [23]. Hypotension may only become apparent late in the disease course, at a point where organ dysfunction is already advanced [22].

Instead, elderly patients with sepsis commonly present with non-specific systemic features that are easily attributed to other causes: acute confusion or delirium, functional decline, falls, reduced oral intake, or simply "not being themselves" as reported by a family member or carer. These presentations are not only atypical but actively misleading; delirium in an elderly patient is frequently attributed to constipation, urinary retention, or a recent medication change before infection is considered [24]. A UK qualitative study of GPs managing serious infections in older adults found that clinicians consistently described difficulty distinguishing infection-related deterioration from functional decline and highlighted the particular challenge posed by patients who cannot give a reliable history [25].

The diagnostic implications for primary care are significant. A GP assessing a frail 82-year-old brought in by their carer because they "seemed off" cannot apply qSOFA thresholds reliably: the patient's baseline blood pressure may already be below 100 mmHg, their baseline cognition may already be impaired, and their respiratory rate may be elevated due to pre-existing lung disease. What the GP must instead rely on is knowledge of that patient's baseline, the trajectory of change over hours to days, and a heightened index of suspicion for infection as a cause of any unexplained acute deterioration in a frail older person. In frail patients, deviation from individual baseline function is often a more clinically meaningful signal than absolute vital sign thresholds; a heart rate of 88 or a temperature of 37.8°C may represent significant physiological stress in a patient whose usual resting values are lower, yet neither would trigger concern using population-based thresholds alone.

Children and Young People

Sepsis in children presents a distinct set of diagnostic challenges in primary care, arising from the need to apply age-specific physiological thresholds, the natural anxiety of parents and carers, and the epidemiological reality that the vast majority of feverish children seen in general practice have self-limiting viral illnesses. Distinguishing the rare child with early bacterial sepsis from the many with uncomplicated infections is one of the most difficult clinical judgements a GP can face.

The Paediatric Early Warning Score (PEWS) was developed and validated in hospital inpatient settings, and its applicability in general practice is variable given the differences in patient acuity, observation frequency, and clinical context. The age-stratified NICE traffic light system for feverish illness in children provides a more transferable framework for risk stratification in community settings, though neither tool was specifically designed for the high-throughput, short-consultation environment of general practice. The newly published NICE guideline NG254 on suspected sepsis in under-16s, released in November 2025, provides updated guidance on recognition and early management in community settings [26]. Alongside this, the Phoenix Sepsis Criteria, published in 2024, offer an internationally validated age-adjusted framework incorporating physiological and laboratory parameters, though their applicability in primary care, where laboratory values are unavailable, remains limited [27].

The cardinal diagnostic principle in children is that parental concern is itself a clinical sign. A parent who reports that their child "looks different" or "is not right" has a diagnostic value that cannot be quantified but should not be dismissed. This is because early behavioural and functional change, reduced interaction, altered feeding, unusual quietness, or irritability, frequently precedes measurable physiological deterioration in children, meaning that parental observation may capture the earliest signs of serious illness before vital sign abnormalities become apparent. The NICE traffic light system enshrines this principle by listing "parental concern" as an amber feature. In primary care, this translates to a low threshold for in-person review, vital sign measurement, and same-day safety netting for any child with a fever and a concerned parent, even when the child appears relatively well at the time of assessment.

Patients With Multimorbidity and Polypharmacy

Patients with multiple long-term conditions occupy a disproportionate share of urgent GP appointments and represent a group in whom sepsis is both more likely to occur and more difficult to recognise. The probability of sepsis following an infection consultation increases substantially with age and frailty [7], and the presence of comorbidities such as diabetes, cancer, chronic kidney disease, and immunosuppressive therapy each independently elevates the risk of serious infection progressing to sepsis.

The diagnostic challenge in multimorbid patients is compounded by polypharmacy. Beta-blockers, among the most widely prescribed medications in general practice for hypertension, heart failure, and atrial fibrillation, attenuate the tachycardic response to physiological stress, masking one of the most accessible early warning signs of sepsis [23]. Corticosteroids suppress the inflammatory response and may prevent fever, while diuretics can cause baseline electrolyte abnormalities and relative dehydration that confound the clinical picture [28]. In a patient on multiple long-term medications with a complex medical history, even an experienced GP may find it difficult to distinguish an acute septic deterioration from decompensation of an existing condition.

Multimorbid patients also carry baseline physiological abnormalities that render standardised scoring systems unreliable. A patient with severe chronic obstructive pulmonary disease may have a resting oxygen saturation of 88% and a respiratory rate of 22 breaths per minute without any acute illness; the same parameters in a previously well patient would constitute red flags. This underscores the importance of knowing the individual patient's baseline, a knowledge that is one of the most valuable and underappreciated assets of the GP who has a longitudinal relationship with their patient population.

Infection-specific diagnostic pitfalls

Three common infection sources in general practice, urinary tract infection (UTI), pneumonia, and skin and soft tissue infection, each carry specific diagnostic pitfalls in the context of sepsis recognition.

UTI is perhaps the most diagnostically treacherous source in elderly patients. The high prevalence of asymptomatic bacteriuria in older women means that a positive urine dipstick result cannot be reliably attributed to a symptomatic UTI. Public Health England guidance revised in 2018 advises against the use of urine dipstick testing as a diagnostic aid in patients over the age of 65, not because dipsticks are universally inappropriate, but because their diagnostic specificity for symptomatic UTI is substantially reduced in this age group due to the high prevalence of asymptomatic bacteriuria, making a positive result unreliable as evidence of active infection [29]. The risk is bidirectional: a positive dipstick may lead to a diagnosis of UTI that is actually asymptomatic bacteriuria, distracting the clinician from the true source of sepsis, while in a genuinely septic patient, the same positive dipstick may prompt a course of oral antibiotics rather than emergency referral, dangerously delaying appropriate treatment. A large population-based cohort study in England demonstrated that the probability of sepsis following a UTI consultation is substantially higher in older and frailer patients, with the number needed to treat (NNT) with antibiotics to prevent one bloodstream infection or sepsis episode falling to 121 in men aged 65-74, compared to substantially higher NNTs in younger and less frail populations [30].

Pneumonia in elderly or frail patients frequently presents without the classical features of cough, fever, and pleuritic chest pain. Confusion, dehydration, reduced mobility, or simply a general deterioration may be the presenting features of a lobar pneumonia in a patient aged over 80. Community-acquired pneumonia (CAP) is one of the most common sources of community-onset sepsis [5], and the absence of respiratory symptoms does not exclude it. A high index of suspicion for lower respiratory tract infection in any frail patient presenting with acute systemic deterioration, combined with pulse oximetry and respiratory rate measurement, is essential.

Skin and soft tissue infections, including cellulitis, represent a frequently underestimated source of sepsis in general practice. Cellulitis may present with erythema and swelling that appears localised, yet systemic features, including tachycardia, fever, and malaise, may indicate early sepsis. In immunocompromised or diabetic patients, the rate of progression from localised cellulitis to systemic sepsis can be rapid, and initial clinical reassurance based on the apparent localisation of the infection can be misleading. Table 2 summarises the atypical presentations and key diagnostic pitfalls associated with each vulnerable patient group discussed in this section.

**Table 2:** Atypical sepsis presentations and diagnostic pitfalls by vulnerable patient group GP: general practitioner; CFS: Clinical Frailty Scale; UTI: urinary tract infection; HR: heart rate; WBC: white blood cell count; CRP: C-reactive protein; SpO₂: oxygen saturation; NICE: National Institute for Health and Care Excellence; COPD: chronic obstructive pulmonary disease; PHE: Public Health England Table adapted from Guz et al. [23] (beta-blocker masking of tachycardia), NICE NG254 [26] (paediatric assessment and parental concern), and Bilsen et al. [31] (urine dipstick guidance in ≥65s).

Patient group	Classic signs often absent	Typical atypical presentation	Common diagnostic traps	Key GP action
Elderly/frail (≥75 years, CFS ≥5)	Fever, tachycardia, classic infective source	Acute confusion/delirium, falls, functional decline, reduced oral intake, "not themselves"	Attributing delirium to constipation, UTI dipstick (asymptomatic bacteriuria), medication change	Compare to baseline; any unexplained deterioration=consider sepsis
Beta-blocker users (any age)	Tachycardia	Presentation of sepsis without compensatory tachycardia; may appear haemodynamically stable	False reassurance from normal heart rate; HR not rising despite physiological stress	Do not use HR as a reassuring sign in beta-blocker users
Immunocompromised (steroids, chemo, biologics)	Fever, raised WBC, CRP elevation	Subtly unwell; infection source may not be localised; markers may be suppressed	Normal inflammatory markers creating false reassurance; low CRP≠no sepsis	Much lower threshold for referral; normal markers do not exclude sepsis
Children (<16 years, NICE NG254)	Localising symptoms	Non-specific fever with parental concern; child "not right"; poor feeding in infants	Reassurance in a well-appearing child; dismissing parental concern	Parental concern is an amber feature; low threshold for in-person review
Multimorbid (COPD) (baseline abnormal observations)	Discriminating vitals	Worsening breathlessness or confusion that is attributed to COPD exacerbation or decompensation	Abnormal baseline observations masking deterioration; SpO₂ 88% "normal for this patient"	Know the patient's baseline; compare to their individual normal, not population thresholds
UTI in the elderly (asymptomatic bacteriuria)	-	Positive dipstick without urinary symptoms; systemic deterioration attributed to UTI	Diagnosing UTI based on dipstick alone in ≥65s; missing the true septic source	Do not use urine dipstick as diagnostic aid in ≥65s (PHE guidance)

Barriers to timely diagnosis in UK general practice

The diagnostic challenges described in previous sections do not occur in isolation. They are compounded by a set of systemic, organisational, and individual barriers that are specific to the UK primary care environment and that collectively reduce the probability of timely sepsis recognition at the community level. Understanding these barriers is a prerequisite for designing interventions that can meaningfully improve outcomes.

Time-Constrained Consultations and the Signal-to-Noise Problem

The average GP consultation in England lasts between 10 and 12 minutes, a duration that has remained largely unchanged despite substantial increases in consultation complexity driven by ageing, multimorbidity, and rising patient expectations [32,33]. Within this time, a GP must take a history, examine the patient, review the record, make a diagnostic and management decision, arrange investigations or referrals, document the encounter, and provide safety netting advice. For most consultations, this is achievable. For the rare consultation involving a patient in the early stages of sepsis, it is a significant constraint.

The fundamental statistical challenge facing every GP is the low prior probability of sepsis within the undifferentiated caseload. Even in a busy practice seeing high volumes of acute presentations, true sepsis is rare compared to the many patients attending with self-limiting viral illness, musculoskeletal pain, or exacerbations of chronic disease. This low base rate means that even a highly sensitive screening tool will generate a substantial number of false positives, while the cost of missing the rare true positive is catastrophic. The cognitive challenge is not simply recognising the septic patient when they present in the classic way, but maintaining a heightened and sustained level of vigilance for the atypical presentation hidden within a stream of patients who are, by and large, not seriously ill.

The Rise of Remote and Telephone Consultations

The COVID-19 pandemic accelerated a structural shift in UK general practice towards remote consultation delivery. By April 2020, an estimated 90% of all primary care consultations were being delivered remotely, predominantly by telephone [34]. While this proportion has since declined, remote consultation has become a normalised and contractually mandated component of GP practice, with all practices required to keep online consultation tools available throughout core hours as of October 2025 [35]. It should be acknowledged that remote consultation also offers potential benefits, particularly for patients who face barriers to attending in person, including those who are housebound, elderly, or geographically isolated, and may facilitate earlier contact with healthcare services than would otherwise occur. However, these access benefits must be balanced against the recognised limitations of telephone and video assessment in detecting clinical deterioration, and escalation pathways and structured safety netting remain essential whenever infection severity is uncertain. The implications for sepsis recognition are significant. Telephone and video consultations necessarily limit the GP's ability to observe the patient's general appearance, assess skin colour and perfusion, measure vital signs, or detect the non-verbal cues, the restlessness, the laboured breathing, and the withdrawn effect, that experienced clinicians rely upon to trigger concern. A practical example is the assessment of respiratory distress: on a video call, a GP may observe that a patient appears breathless, but cannot measure respiratory rate, assess accessory muscle use, or detect central cyanosis, all of which may be critical in distinguishing early sepsis from a self-limiting respiratory illness. Mitigation strategies include maintaining a low threshold for in-person review when red flags are unclear on remote assessment, ensuring all remotely triaged patients with possible infection receive explicit safety netting instructions, and having clear practice protocols for escalating patients who cannot be adequately assessed by telephone or video. A UK cohort study using Clinical Practice Research Datalink (CPRD) data found that telephone consultations were associated with a significantly higher odds of potentially missed acute deterioration compared with face-to-face consultations and that each additional five minutes of consultation time was associated with a 10% reduction in the odds of a self-referred hospital admission, a proxy for missed deterioration [36]. These findings are of direct relevance to sepsis: the patients most likely to deteriorate rapidly with sepsis are among those most difficult to assess by telephone.

Training and Awareness Gaps

Despite more than a decade of national sepsis awareness campaigns, training programmes, and guideline development, gaps in GP knowledge and preparedness persist. A 2021 survey of GP practices in Cornwall found marked variation in sepsis preparedness: approximately one-fifth of surgeries reported that not all clinical staff were familiar with the concept of Red Flag Sepsis, and in just over a third of practices, no receptionist training on the topic had been delivered [4]. The Care Quality Commission (CQC) includes sepsis preparedness within its inspection framework for GP practices, specifically under the "being safe" quality statement [37], yet the survey findings suggest that this has not driven uniform compliance.

The NHS e-learning programme on sepsis in primary care, developed in partnership with NICE and updated to reflect the 2024 guidance, provides a freely available resource for GP teams [38]. However, engagement with optional e-learning in an already pressured workforce is variable, and completion does not guarantee behavioural change in the consulting room. A 2024 British Medical Association (BMA) poll of GP registrars found that 66% reported working outside scheduled hours most or every day [32], while a 2025 General Medical Council (GMC) report found that nearly half of all GPs cited workload as their greatest professional challenge [39]. In this context, time available for professional development and training is limited.

Systemic and Organisational Factors

Beyond the individual consultation, several systemic factors limit the GP's ability to act on concerns about possible sepsis. Access to same-day investigations, blood tests, chest radiography, and urine culture, varies significantly between practices and localities, and the results of investigations ordered during a GP consultation are often not available for several hours or the following day. For a patient with suspected early sepsis, this delay is clinically unacceptable; the decision to refer must often be made on clinical grounds alone, without laboratory support.

Ambulance response times and the capacity of receiving emergency departments to accept sepsis referrals also affect the downstream consequences of GP decision-making. A GP who calls 999 for a patient with possible sepsis may face a prolonged wait for an ambulance or may be advised to arrange transport independently. In rural or remote areas, transfer times to the nearest emergency department may exceed an hour, creating a window of time in which the patient's condition may deteriorate without access to definitive treatment. The updated NICE guidance explicitly addresses this scenario, recommending that mechanisms be in place for GPs to administer antibiotics to high-risk patients in remote areas where transfer time to an emergency department exceeds one hour [10].

Finally, the fragmentation of care, between daytime GP services, out-of-hours providers, NHS 111, and emergency departments, creates information gaps that increase the risk of missed or delayed diagnosis. A patient who contacts NHS 111 in the evening and is advised to see their GP the following morning may deteriorate overnight. A telephone triage cohort study of over 155,000 patients presenting to out-of-hours GP cooperatives found that approximately one-third of patients who subsequently developed adverse sepsis-related outcomes were not referred to hospital after their first contact with healthcare services [40]. The strongest predictors of adverse outcome in that study were age, the type of contact (home visit versus clinic), and presenting complaints including general malaise and shortness of breath, precisely the non-specific features most likely to be underweighted in a brief telephone triage conversation.

Practical recommendations for UK GPs

**Figure 1:**
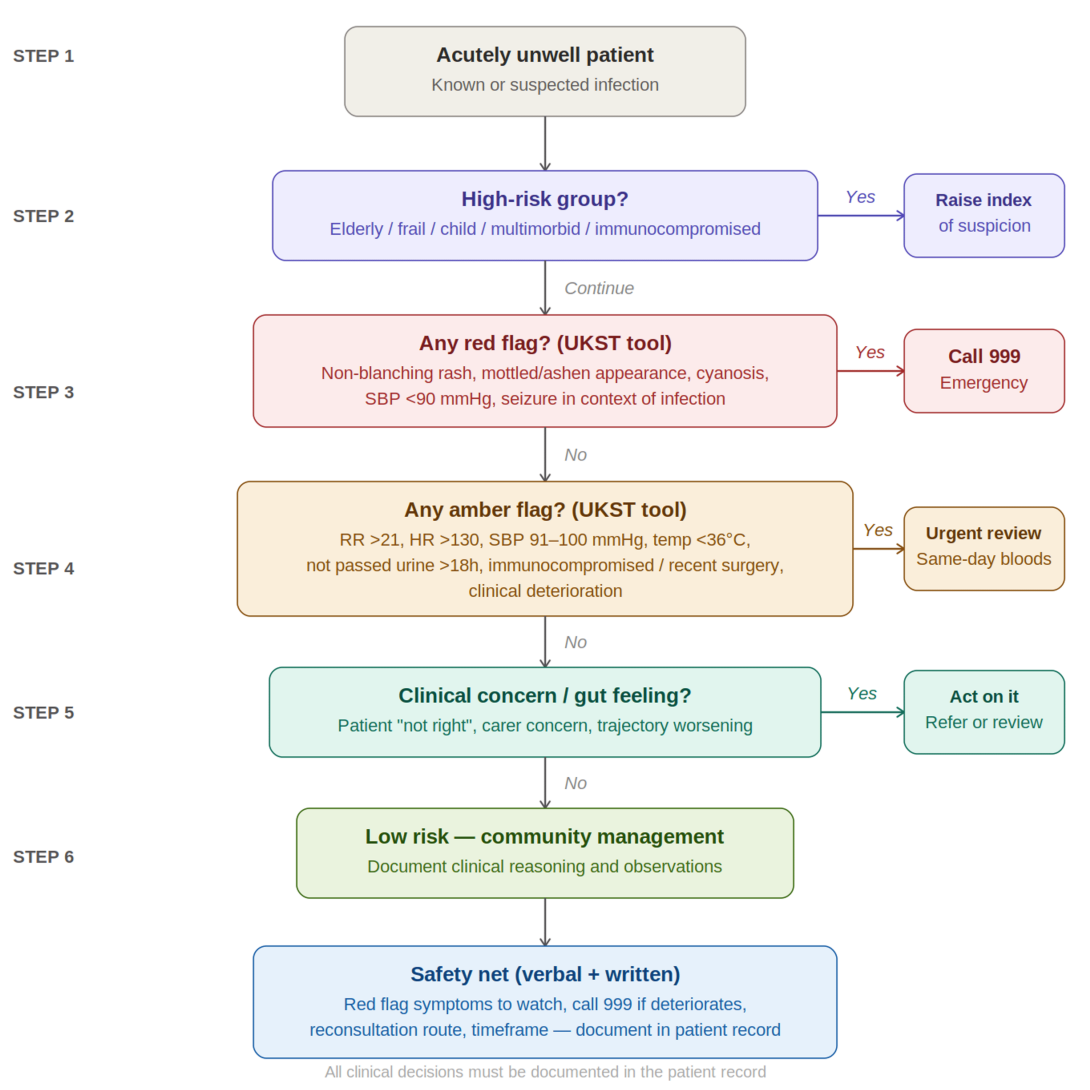
A structured approach to sepsis risk assessment in UK general practice UKST: UK Sepsis Trust; SBP: systolic blood pressure; RR: respiratory rate; HR: heart rate; SpO₂: oxygen saturation; temp: temperature; NICE: National Institute for Health and Care Excellence The pathway guides the clinician from initial presentation through sequential risk stratification using the UKST primary care tool criteria [17] and NICE NG253 [10]. Red flag criteria prompt immediate emergency referral (999). Amber flag criteria prompt urgent same-day review. Clinical concern or gut feeling, even in the absence of formal flags, should prompt referral or review in keeping with UKST guidance [17]. Patients assessed as low risk should receive structured verbal and written safety netting before discharge. All clinical decisions must be documented in the patient record. This pathway is intended as a supportive framework for clinical reasoning and structured documentation. It has not been prospectively validated as a diagnostic algorithm and should be used to supplement, not replace, clinical judgement. The figure was created by Omar Elbaroumi using Inkscape (Inkscape Project, inkscape.org), an open-source vector graphics editor, and exported as a PNG image file.

The preceding sections have established that sepsis recognition in primary care is compromised by a combination of ill-fitting diagnostic tools, atypical presentations in vulnerable patients, and systemic barriers to timely assessment. The following recommendations are grounded in current evidence and guideline requirements and are intended to be practically applicable within the constraints of UK general practice (Figure 1).

1. Adopt and Display the UK Sepsis Trust Primary Care Tool

The UK Sepsis Trust primary care screening tool, updated in 2024 in alignment with NICE guidance, represents the most contextually appropriate structured approach to sepsis risk stratification currently available for GP settings [17,41]. It should be displayed visibly in consulting rooms and treatment rooms, made available as an electronic reference in clinical systems, and used to structure documentation when sepsis is considered. Crucially, the tool should be understood as a support for clinical judgement, not a replacement for it: the UK Sepsis Trust tool itself states that any patient in whom there is clinical concern should be referred, regardless of whether formal red or amber flags are met [41]. GPs should familiarise themselves with the current version and check for updates as NICE guidance evolves.

2. Trust and Document Clinical Concern

The evidence consistently demonstrates that GP clinical intuition, while informal, is diagnostically valid and broadly tracks the physiological parameters that define sepsis risk [9]. When a GP has a gut feeling that a patient is seriously unwell, that feeling should be acted upon and documented. From both a patient safety and medicolegal perspective, documenting clinical reasoning, including the absence of red flags when present, is as important as documenting positive findings. A British Journal of General Practice cross-sectional study found that GPs frequently do not document the safety netting advice they give verbally, with significant medicolegal implications in the event of adverse outcomes [42]. GP clinical systems include structured templates for physiological observations in acutely unwell patients; these should be used routinely whenever sepsis is considered, even when the patient is ultimately assessed as low risk.

3. Apply Heightened Vigilance in High-Risk Groups

For elderly, frail, and multimorbid patients presenting with any acute deterioration, infection should be considered as a potential cause until excluded. In this group, the absence of classical signs, fever, tachycardia, and a clear infective source, does not reduce the probability of sepsis. The relevant clinical questions are: Is this patient's current state different from their baseline? Is there any identifiable source of infection? Could this be sepsis? For patients on beta-blockers, the absence of tachycardia is not reassuring. For those on corticosteroids, the absence of fever is not reassuring. For those with cognitive impairment, acute confusion or behavioural change should raise immediate concern for an underlying infection with systemic features [22,23].

For children presenting with fever and parental concern, the threshold for in-person review should be low. Even when the child appears relatively well at presentation, parental concern is a validated amber feature under NICE guidance and should not be dismissed [26]. Same-day safety netting with a clear reconsultation plan and verbal and written information about red flag signs is mandatory for all children assessed for possible sepsis who are managed in the community.

4. Safety Net Rigorously and Documentably

Safety netting is both a clinical and medicolegal imperative in all consultations where sepsis is considered and the patient is not referred [43,44]. Best practice requires verbal and written safety netting advice that specifies which symptoms should prompt urgent reconsultation (including non-blanching rash, significant deterioration, inability to stand, confusion, or breathlessness at rest), a clear and accessible route for reconsultation (including out-of-hours contacts and 999), and an appropriate timeframe. A realist review of safety netting in primary care identified 15 recommendations for effective communication, emphasising that advice should be tailored to the individual patient, verified for understanding, and documented with sufficient detail to support continuity of care [44]. Standardised sepsis patient information leaflets, available from the UK Sepsis Trust and Royal College of General Practitioners, should be provided where possible [45].

5. Ensure Practice-Level Preparedness

Sepsis preparedness is the responsibility not only of individual GPs but of practices. Every practice should have an identified sepsis lead, all clinical staff should be familiar with Red Flag Sepsis, and reception and administrative staff should be trained to escalate patients who describe symptoms consistent with possible sepsis, including severe illness in a young person, a non-blanching rash, or a carer reporting that their relative is significantly worse than usual [4,45]. The Royal College of General Practitioners sepsis toolkit provides resources for practice-level training, audit, and quality improvement [45].

The NHS e-learning programme on sepsis in primary care, freely available through the Learning Hub, should be completed by all GP clinical staff and updated with reference to current NICE guidance [38]. A systematic review of sepsis education interventions found that active learning approaches, including simulation and case-based discussion, produced greater and more durable gains in knowledge and confidence than didactic teaching alone [46]. Primary care networks and integrated care boards should consider commissioning in-person simulation sessions focused on sepsis recognition in primary care, particularly emphasising atypical presentations in the elderly, frail, and children [47].

6. Exercise Caution With Remote Consultations for Acutely Unwell Patients

The evidence linking telephone consultations to a higher odds of missed acute deterioration [36] supports a policy of a lower threshold for face-to-face review in patients triaged as acutely unwell with possible infection. For any patient where sepsis is a possibility based on the telephone or online triage, in-person assessment with measurement of vital signs, particularly respiratory rate, pulse, blood pressure, and oxygen saturation, should be strongly considered before a remote management plan is enacted. When remote management is unavoidable, safety netting must be explicit, accessible, and documented, and the patient or carer must be given a direct and clear instruction to call 999 if there is any deterioration.

Key take-home points for UK GPs

Sepsis most commonly originates in the community, yet the diagnostic tools available to GPs were developed and validated in hospital settings, a fundamental mismatch that drives missed diagnoses at the community level.

Classical sepsis signs (fever, tachycardia, obvious infective source) are frequently absent in the patients most at risk, the elderly, frail, children, and those with multimorbidity. Deviation from individual baseline function is often more informative than absolute vital sign thresholds.

A negative qSOFA score does not rule out sepsis and should never be used to provide false reassurance in primary care.

The UK Sepsis Trust primary care tool is the only structured framework specifically designed for GP settings and is freely available, yet fewer than 2% of GPs report using it.

Beta-blocker therapy may mask tachycardia; corticosteroids may suppress fever. The absence of these classical signs in patients on these medications is not reassuring.

Safety netting is both a clinical and medicolegal imperative for every consultation in which sepsis is considered and the patient is not referred. It must be verbal, written, and documented.

Given that telephone and video consultations carry a higher risk of missed acute deterioration, clinicians should maintain a low threshold for in-person review whenever infection severity is uncertain.

Parental concern in a feverish child is a validated amber feature and should never be dismissed; early behavioural change frequently precedes measurable physiological deterioration.

All practices should have a named sepsis lead, trained reception staff, and a clear escalation protocol.

Limitations

This review has several limitations that should be acknowledged. As a narrative rather than systematic review, the literature search was not exhaustive and was not pre-registered; the selection of studies reflects the author's judgement rather than a predetermined protocol, and publication bias cannot be excluded. The majority of primary care-specific evidence identified, including the most relevant diagnostic tool validation studies, originates from Dutch out-of-hours and home-visiting services, which differ substantially from the daytime UK GP surgery context in terms of patient acuity, visit type, and point-of-care resource availability. Extrapolation of these findings to UK general practice should therefore be made with caution. Additionally, the evidence base for sepsis recognition in primary care remains sparse overall, and several recommendations in this review are necessarily informed by expert consensus, guideline committee opinion, and indirect evidence from emergency department or hospital settings rather than high-quality primary care trials. These limitations reflect the state of the field rather than shortcomings specific to this review and themselves underscore the central argument: that primary care-specific sepsis research is urgently needed.

## Conclusions

Sepsis recognition in UK general practice remains one of the most demanding diagnostic challenges in primary care medicine. The difficulty is not simply one of awareness or training, but reflects a deeper structural mismatch: the tools and frameworks developed to identify sepsis were built for hospital settings, while the majority of cases originate in the community, where the clinical environment is fundamentally different. The patients most at risk, the elderly, the frail, children, and those with multiple comorbidities, are also those whose presentations most frequently deviate from the classical sepsis phenotype and in whom the absence of fever, tachycardia, or an obvious infective source does not reduce the probability of sepsis but increases the likelihood of delayed recognition. The GP's longitudinal knowledge of the patient, their baseline, and their trajectory is an irreplaceable diagnostic asset that no scoring system can replicate, and this review argues that it should be recognised, structured, and supported, not replaced by adapted hospital tools that were never validated for primary care populations.

The available evidence points to several clear priorities. The UK Sepsis Trust primary care tool, the only structured framework specifically designed for community use, must achieve far greater implementation, provided that its current awareness rate of fewer than 12% among GPs is not acceptable given the patient safety challenge it addresses. Safety netting must be treated as a clinical and medicolegal standard for every consultation in which sepsis is considered, and the shift towards remote and telephone consultation requires urgent mitigation given the evidence linking it to missed acute deterioration. At the system level, prospective UK-based studies of sepsis recognition in daytime general practice are urgently needed to develop and validate diagnostic tools fit for the GP consulting room. Finally, the regulatory and medicolegal environment must be proportionate: supporting GPs with better tools, better training, and better systems, rather than primarily responding to failure through litigation, is the most effective route to reducing preventable sepsis mortality in the community.
